# P-1057. Leveraging A Global-Scale Atlas of Phage Host Interactions to Discover Novel Precision Antimicrobials

**DOI:** 10.1093/ofid/ofae631.1246

**Published:** 2025-01-29

**Authors:** Sam Bryson, Benjamin Auch, Jonas Grove, Bradley J Nelson, Demi Glidden, Emily Reister, Ivan Liachko

**Affiliations:** Phase Genomics, Inc., Seattle, Washington; Phase Genomics, Inc., Seattle, Washington; Phase Genomics, Inc., Seattle, Washington; Phase Genomics, Inc., Seattle, Washington; Phase Genomics, Inc., Seattle, Washington; Phase Genomics, Inc., Seattle, Washington; Phase Genomics, Inc., Seattle, Washington

## Abstract

**Background:**

A growing global antimicrobial resistance (AMR) crisis fueled by the overuse of traditional antibiotics has created an urgent need not only for new antimicrobials, but entirely new approaches for sustained therapeutic development against existing and emerging pathogens. Endolysins (lysins) have demonstrated both efficacy and safety as precision antimicrobials that overcome the resistance problem posed by most other antibiotic modalities. However, due to the difficulty of identifying new sources of lysins, development has been slow.

**Methods:**

We have adapted a unique genomic technology, proximity ligation sequencing, to harness the vast genetic diversity of microbial viruses (phages) to discover large-numbers of protein-based interventions against microbes. Proximity-guided metagenomics allows us to pinpoint the microbial targets of phages *in situ*, recovering the genomes of both microbe and phage without culturing or isolation of either party. Inherently, each lytic phage genome encodes one or more more proteins (endolysins) which can lyse their host cell. Each of these proteins represents a powerful tool in the targeted destruction of a selected organism, and because we know which species of microbe each phage infects, we can identify candidate lysins from proximity-guided metagenomic data alone.

**Results:**

As part of our global-scale metagenomics project, we used our ProxiMeta platform to recover the target-resolved genomes of hundreds of thousands of phages across the tree of life. We will share published and unpublished work describing our discovery and evaluation of these lysins, and their applications in selectively killing microbes involved in human disease.

**Conclusion:**

Phage genomes from metagenomic databases are a rich source of antimicrobial proteins that can be developed into novel antimicrobial agents.

**Disclosures:**

**All Authors**: No reported disclosures

